# How robust is familiar face recognition? A repeat detection study of more than 1000 faces

**DOI:** 10.1098/rsos.170634

**Published:** 2018-05-30

**Authors:** Angus F. Chapman, Hannah Hawkins-Elder, Tirta Susilo

**Affiliations:** 1 School of Psychology, Victoria University of Wellington, Wellington 6040, New Zealand; 2 Department of Psychology, University of California San Diego, La Jolla, CA, USA; 3 ARC Centre of Excellence in Cognition and its Disorders, Sydney, New South Wales, Australia

**Keywords:** face recognition, face memory, familiarity, repeat detection

## Abstract

Recent theories suggest that familiar faces have a robust representation in memory because they have been encountered over a wide variety of contexts and image changes (e.g. lighting, viewpoint and expression). By contrast, unfamiliar faces are encountered only once, and so they do not benefit from such richness of experience and are represented based on image-specific details. In this registered report, we used a repeat detection task to test whether familiar faces are recognized better than unfamiliar faces across image changes. Participants viewed a stream of more than 1000 celebrity face images for 0.5 s each, any of which might be repeated at a later point and has to be detected. Some participants saw the same image at repeats, while others saw a different image of the same face. A post-experimental familiarity check allowed us to determine which celebrities were and were not familiar to each participant. We had three predictions: (i) detection would be better for familiar than unfamiliar faces, (ii) detection would be better across same rather than different images, and (iii) detection of familiar faces would be comparable across same and different images, but detection of unfamiliar faces would be poorer across different images. We obtained support for the first two predictions but not the last. Instead, we found that repeat detection of faces, regardless of familiarity, was poorer across different images. Our study suggests that the robustness of familiar face recognition may have limits, and that under some conditions, familiar face recognition can be just as influenced by image changes as unfamiliar face recognition.

## 1. Introduction

The ability to recognize faces is crucial to successful functioning in the social environment. Our ability to recognize hundreds or even thousands of faces points to the existence of a sophisticated face recognition system. To study how this system works, scientists typically use two kinds of stimuli: *familiar* faces and *unfamiliar* faces. Many basic signatures of face processing apply to both types of stimuli, including perceptual markers like the face inversion effect ([1], but see [2]), the composite face effect [3], the contrast negation effect [4], the Thatcher effect [5] and the face aftereffect [6,7], as well as brain markers from fMRI (activation in fusiform face area) [8], EEG (N170) [9] and MEG (M170) [10]. These findings show that some processes in face recognition are fundamental and operate on all faces regardless of familiarity.

However, a great deal of evidence also shows that familiar and unfamiliar faces are processed in different ways. Familiar faces are recognized better and quicker than unfamiliar faces [11,12]. Familiar faces are better recognized using internal features (e.g. eyes and mouth) than external features (e.g. hair) [12–15]. Familiar faces are less likely to be missed in change detection and attentional blink paradigms [16,17]. Finally, familiar and unfamiliar face recognition doubly dissociate – some acquired prosopagnosics are impaired with familiar but not unfamiliar faces [18,19], while others show the opposite pattern [20].

Taken together, these findings motivate the idea that familiar and unfamiliar faces rely on distinct mental representations [21–23]. Familiar faces are those we have seen many times in a wide range of contexts (e.g. different expression, viewing angle and lighting condition). Familiar faces are thought to rely on *structural codes*, which reflect some sort of averaging process in which a robust representation of a face is constructed across multiple encounters that extracts the underlying stability of a face while filtering out irrelevant variability [11,24,25]. Mental representations of familiar faces are therefore rich and encompassing because they integrate diverse experiences to create a stable impression of a specific person. On the other hand, unfamiliar faces are encountered only once, which means their representations can only comprise the one exposure. Unfamiliar faces are said to rely on *pictorial codes*, which involve storing the visual details of a particular face image [11]. Unfamiliar face recognition is therefore more analogous to image matching, and as a result changes in specific image details typically lead to poor performance.

The distinction above predicts that changes to images will impair recognition of familiar faces less than unfamiliar faces. Consistent with this idea, many studies have found that compared to unfamiliar faces, familiar faces are more impervious to a wide variety of image changes including expression [26,27], lighting [28], viewpoint [29], head orientation [26,27], image quality [30,31] and environmental context [32]. However, these studies tend to employ traditional tasks such as the *old/new recognition task* (presenting a subset of stimuli from the ‘study’ phase interspersed with novel stimuli and asking participants to make an ‘old/new’ judgement) [12,26,33,34], *array face-matching task* (asking participants to match a given target face to a different image of the target from a multiple-face array containing an image of the target and a number of novel face images) [2,35] or *paired face-matching task* (presenting participants with pairs of faces and asking them to make a ‘same/different’ judgement for each pair) [2,13,35]. Unlike real-world face learning, these tasks are typically short in duration (between 5 and 20 min) and use a relatively small number of stimuli (between 30 and 150) [2,12,26,33]. This means that we do not really know whether familiar face recognition remains robust to image changes under more demanding experimental conditions with significant memory load.

Here, we investigate whether familiar face recognition remains robust to image changes with a challenging repeat detection task [36–38]. Our task uses more than 1000 face images and takes almost 1 h to complete. In this task, participants view a stream of face images and respond whenever they detect a repeated face. Participants do not know which faces will be seen again, so they must attend to and memorize every face as a potential target. This task is similar to those used in studies of repetition priming [39,40], in which presentation of face images enhances recognition of those faces on a later task. We use celebrity faces as stimuli to avoid the practical issues of devising a stimulus set that is specific to each participant (e.g. friends or family members). We perform a familiarity check at the end of the experiment to assess each participant's familiarity with a subset of the stimuli and to classify which faces are familiar and which are not [13].

**Figure 1.**
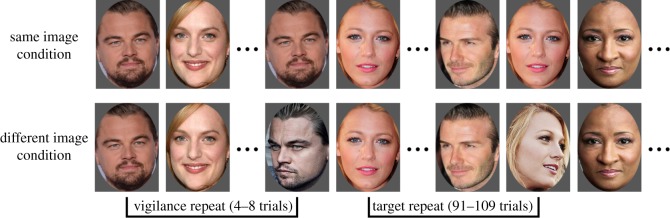
Trial design of the repeat detection task. Each face image was shown for 500 ms with a 1000 ms inter-trial interval. Participants pressed the space bar when they detect repeats.

Our critical manipulation involves the type of image shown for repeated faces (figure 1). Repeated images could appear at one of two different times: *vigilance repeats* occurred shortly following the initial presentation of a face, and were included to assess participants' engagement with the task; *target repeats* occurred much longer after the initial face was presented, and were the main concern of our analysis. In one condition, the repeats show the same image as the initial presentation, while in the other condition the repeats present different images of the same person. Our prediction is threefold. First, we expect better repeat detection when the target face is familiar rather than unfamiliar, regardless of image type. Second, we expect better detection when the target image is the same as the initial image than when it is a different image of the same face, regardless of familiarity. Third, we expect that detection of familiar faces will be less influenced by image type than detection of unfamiliar faces is, and that detection of unfamiliar faces will be poorer when the target image is different from the initial face.

## 2. Method

### 2.1. Pilot study

We conducted a pilot study to estimate the effect of familiarity on repeat detection of faces. The full method and results are provided in the electronic supplementary material. There was a familiarity by image interaction, *F*_1, 43_ = 5.79, *p* = 0.020, $\eta_{p}^{2}\;=0.119$, BF_10_ = 2.84, which revealed that unfamiliar faces were better detected with ‘same’ rather than ‘different’ images, *t*_42.77_ = 3.76, *p* < 0.001, *d_s_* = 1.15, BF_10_ = 52.10, while familiar faces were detected equally well regardless of image, *t*_41.28_ = 0.99, *p* = 0.327, *d_s_* = 0.30, BF_10_ = 0.44.

### 2.2. Power analysis

No studies have compared familiar and unfamiliar faces using a repeat detection task, so we conducted power analysis based on the results of the pilot study. For the interaction effect, we converted $\eta_{p}^{2}$ (0.119) to $\omega_{p}^{2}$ (0.096) to reduce bias in estimating effect size [41], which corresponded to Cohen's *f* = 0.326. A power analysis performed in GPower 3.1 with an effect size of 0.294 (which is 90% of the pilot effect size, to account for potential overestimation) gives a required sample size of 124 to achieve 90% power. Power analyses for the main effects of familiarity and image produced smaller required samples (main effect of familiarity: Cohen's *f* = 2.145, *n* = 6; main effect of image: Cohen's *f* = 0.311, *n* = 112). This means a sample size of 124 will have greater than 90% power to test our predictions and capture all three effects (should they exist). We therefore planned to test participants until a sample of 124 non-excluded participants was reached.

We note that our effect size for the interaction between familiarity and image is smaller than those reported in past studies. For example, the effect sizes ($\eta_{p}^{2}$) for the interaction between familiarity and image were 0.27 and 0.22 in Armann *et al*. [33], respectively, and 0.58 in Bruce [26] (Experiment 2). The effect size discrepancy between these studies and ours is likely due to task and/or stimulus differences, and possibly publication bias.

### 2.3. Participants

One hundred and forty-six first-year psychology students at Victoria University of Wellington participated for course credit. They were randomly assigned to the ‘same’ image (*n* = 73) or ‘different’ image (*n* = 73) conditions. Five participants (two in the ‘same’ condition and three in the ‘different’ condition) did not complete the familiarity check due to a technical error, and their data were excluded. Seventeen participants (nine in the ‘same’ condition and eight in the ‘different’ condition) were excluded based on pre-determined criteria (see Analytic protocol). Our final sample therefore consisted of 124 participants, equally assigned to each condition. Participants were between 18 and 27 years of age (*M* = 18.71, s.d. = 2.10) and were predominantly women (*n* = 98). Most reported their ethnicity as New Zealand European/Pākehā (*n* = 99), with the remaining Māori (*n* = 11), Pacific (*n* = 1), Asian (*n* = 4) or as another unspecified ethnicity (*n* = 9).

### 2.4. Materials

To create our stimulus set, we first drafted a list of 1006 celebrities who were actors, musicians or other notable figures in popular culture. For each celebrity, we downloaded several face images from a Google Image search. Two images were selected for each celebrity to make up the stimulus set. The two images for each celebrity varied in terms of lighting, viewpoint, expression and date taken. Images were cropped in an oval to exclude non-face, background information (as in e.g. [37,38]). Each face was coded on the four demographic attributes: glasses (4.7% of faces); skin colour (10.1% dark, 89.9% light); gender (53.7% male, 46.3% female); and age (0.8% child, 91.6% adult, 7.6% elderly). We selected 100 celebrities as target repeats with a demographic breakdown that mirrors that of the full stimulus set to ensure that targets and non-targets cannot be easily discriminated using demographic clues. We also selected 25 extra celebrities from the remaining stimulus set to use as vigilance repeats.

### 2.5. Procedure

#### 2.5.1. Repeat detection task

Participants viewed a stream of 1131 images, each presented for 500 ms with a 1000 ms inter-trial interval. Of these images, 125 depicted a famous face shown earlier in the experiment, and participants were instructed to press the space bar when they detected these repeats. There are three types of trials. The first are *vigilance repeats* (25 trials), in which a face was repeated quickly, within four to eight trials of the initial presentation. These were included to gauge whether participants were focusing on the task. The second are *target repeats* (100 trials), in which a face was repeated, but the number of intervening faces was greater, within 91–109 trials of the initial presentation. The third are *fillers* (1006 trials) that include presentations of faces which were shown only once during the task (881 trials), but also the initial presentation of a target face in repeat trials (125 trials). For both *vigilance* and *target repeats*, the correct response for participants was to press the space bar, while for *fillers* the correct response was no response. Figure 1 shows our trial design. Participants were given a 30-s rest break every 162 images (approximately 4 min).

The critical manipulation was whether the repeated face (for both vigilance and target repeats) was the exact same image as the initial face (‘same’ condition), or whether it was a different image of the same face (‘different’ condition). This was manipulated between subjects. The first image presented was randomly selected from the two images created per celebrity (see Materials), while the second image was determined by condition.

#### 2.5.2. Familiarity check

Following the repeat detection task, we asked participants to identify 100 target faces and 100 demographically matched filler faces. Participants were presented with each face image and asked to identify the person by typing their name in a text box. The face images shown were the same as the first presentation of the target in the repeat detection task. Participants were instructed that a sentence describing the famous face (e.g. a character's name) was also acceptable. No time or word limit was placed on these responses. Responses were classified as ‘familiar’ if participants provided the correct full name, made a minor error in naming, gave a specific example that identified the individual (e.g. a character played in a TV show or film), or provided a general category (e.g. actor, singer) that the famous individual belonged to. Responses that were blank or otherwise incorrect were classified as ‘unfamiliar’. All non-blank responses (*n* = 7771) were scored by two coders (*κ* = 0.931) and any disagreements were resolved through discussion. In addition to identifying each of the target faces, participants were asked to rate their familiarity with the face on a 7-point Likert scale, from 1 (not at all familiar with this person) to 7 (highly familiar with this person).

### 2.6. Analytic protocol

The analytic protocol described here was as used in our pilot study.

#### 2.6.1. Exclusion

Participants were excluded based on specific criteria for both familiarity and repeat detection tasks. We excluded data at the subject level, but not at the level of individual trials. *Repeat detection task:* We first excluded participants based on vigilance performance. Bainbridge and colleagues [37] excluded participants who identified less than half of any 10 consecutive vigilance repeats. In our study, because vigilance repeats are more difficult for the ‘different’ group, we adjusted this procedure to avoid excluding unequal numbers of participants in each group. We excluded participants whose vigilance accuracy was greater than 2 s.d.s below the mean for their condition. *Familiarity check:* Our analysis requires that participants are familiar with some target faces, so we also excluded participants who were familiar with less than 10% of target faces.

#### 2.6.2. Data checks

To confirm data quality, we performed four data checks by testing whether the number of: (i) familiar faces identified differed between groups, (ii) hits for vigilance repeats was higher than false alarms for fillers, (iii) hits for vigilance repeats was higher for ‘same’ than ‘different’ groups, and (iv) false alarms for fillers was comparable across groups.

#### 2.6.3. Main analysis

All analyses were conducted in R [42]. For the main analysis assessing familiarity on repeat detection for the two groups, we fit a linear mixed-effects model with the package *lme4* [43], and analysis of variance was conducted with *lmerTest* [44] using Type III sums of squares and Satterthwaite approximation for degrees of freedom. To test our hypothesis that familiarity will influence detection of familiar and unfamiliar faces differently depending on the type of image, we conducted a 2 × 2 mixed ANOVA with image (same versus different) and familiarity (familiar versus unfamiliar, based on the responses of each participant during the familiarity check) as fixed factors, with a random intercept modelled for each participant. Because the number of faces people recognize as familiar and unfamiliar are unlikely to be equal, a weighted contrast was used so that means reflect the true average across this factor. Analysis of null results was complemented by Bayesian statistics. Using the *BayesFactor* package [45], we conducted Bayesian *t*-tests alongside the traditional *t*-tests; *F*-ratios were first converted to *t*-values [46]. We report the Bayes factor in favour of the alternative hypothesis (BF_10_) based on analysis using a ‘default’ prior with scale = 0.707 [47].

## 3. Results

### 3.1. Registered analyses

#### 3.1.1. Data checks

**Figure 2.**
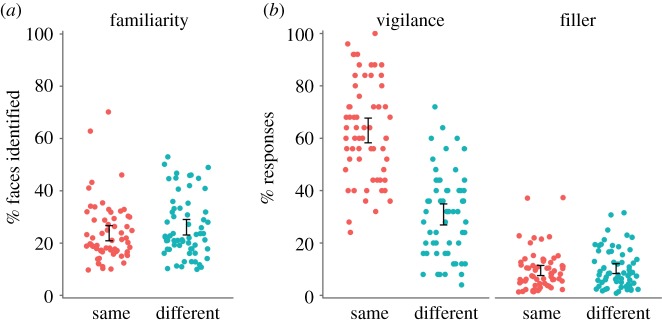
Individual data for data checks. Dots are jittered horizontally to improve visibility. The left panel shows the number of familiar faces is comparable across groups. The right panel shows that hit rates for vigilance repeats are higher for ‘same’ rather than ‘different’ groups, whereas false alarms for fillers are similar. Error bars show 95% CI around the mean.

To ensure that data from the familiarity and repeat detection tasks were valid for further analysis, we conducted four checks. First, in the familiarity check, participants identified a reasonable number of faces (*M* = 24.9%, s.d. = 11.6, range = 10.0–70.0), which did not differ between groups, *t*_121.94_ = 1.10, *p* = 0.273, *d* = 0.20, BF_10_ = 0.332. Second, in the repeat detection task, hit rates for vigilance repeats (*M* = 46.9%, s.d. = 23.6) were higher than false alarms for fillers (*M* = 9.9%, s.d. = 7.5), *t*_123_ = 18.31, *p* < 0.001, *d* = 1.64, BF_10_ > 1000. This shows that performance on vigilance trials was not simply due to participants responding at random. Third, vigilance detection was greater with ‘same’ rather than ‘different’ images, *t*_119.12_ = 10.34, *p* < 0.001, *d* = 1.87, BF_10_ > 1000, consistent with previous literature [26,33,38]. Four, false alarms for fillers did not differ between groups, *t*_121.94_ = 0.54, *p* = 0.591, *d* = 0.10, BF_10_ = 0.219, showing that false responses to *fillers* were unaffected by changes to the *target* images. Table 1 contains group means and s.d. for data checks; figure 2 plots individual data. These data checks produced similar results as were found in the pilot study.

**Table 1.** Group mean (s.d.) percentages of faces identified in the familiarity check, hit rates for vigilance repeats and false alarms for fillers in the repeat detection task.

group	familiarity check % faces identified	repeat detection task vigilance	fillers
same	23.8 (11.4)	63.0 (18.6)	9.5 (7.5)
different	26.0 (11.7)	32.8 (12.2)	10.2 (7.4)

#### 3.1.2. Main analysis

**Figure 3.**
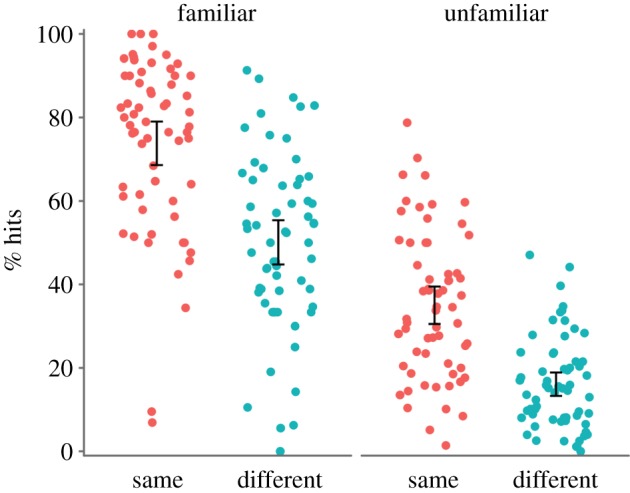
Individual data for correct detection of target repeats by familiarity and group. Dots are jittered horizontally to improve visibility. Familiar faces were detected better than unfamiliar faces in both groups, and faces were detected better by the ‘same’ rather than by the ‘different’ group. Error bars show 95% CI around the mean.

We compared detection hit rates in each group for target faces that participants reported as familiar and unfamiliar. The results are presented in table 2 and figure 3. Familiar faces were detected more accurately (*M* = 61.9%) than unfamiliar faces (*M* = 25.5%), reflected by the main effect of familiarity, *F*_1, 122_ = 595.74, *p* < 0.001, $\eta_{p}^{2}\;=0.830$, BF_10_ > 1000. There was also a main effect of image, *F*_1, 122_ = 55.44, *p* < 0.001, $\eta_{p}^{2}\;=0.312$, BF_10_ > 1000, as repeat detection was better for the ‘same’ (*M* = 44.7%) rather than the ‘different’ (*M* = 24.6%) images. However, unlike in the pilot study, there was no interaction between familiarity and image, *F*_1, 122_ = 2.60, *p* = 0.110, $\eta_{p}^{2}=0.021$, BF_10_ = 0.617, although Bayes factors provided inconclusive evidence for this null effect. Unfamiliar faces were better detected with ‘same’ rather than ‘different’ images, *t*_102.59_ = 7.15, *p* < 0.001, *d* = 1.30, BF_10_ > 1000, and this was also true for familiar faces, *t*_121.97_ = 6.37, *p* < 0.001, *d* = 1.15, BF_10_ > 1000.

**Table 2.** Group mean (s.d.) hit rates for detection of familiar and unfamiliar targets in the repeat detection task.

	% hits	
group	familiar faces	unfamiliar faces
same	73.8 (20.6)	35.0 (17.6)
different	50.1 (20.9)	16.1 (11.1)

Could the results be accounted for by the fact that the ‘different’ image condition is more difficult than the ‘same’ image condition? To address this issue, we restricted our analysis to a subset of participants in each condition such that they were equated on vigilance performance. To achieve this, we used an iterative approach where we selected the *n* participants in the ‘different’ image condition who performed best on vigilance repeats, and the *n* participants in the ‘same’ image condition who performed worst on vigilance repeats. We varied *n* across the range of the sample (*n* = 2–62 in each condition) to find the point at which the difference in vigilance performance between groups was minimized. This point was reached by selecting the top 27 participants in the ‘different’ image condition, and the bottom 25 participants in the ‘same’ image condition. This subset was equated on vigilance performance (*M* = 45.2%, s.d. = 9.5), *t*_49.99_ = 0.26, *p* = 0.799, *d_s_* = 0.07, BF_10_ = 0.286; however, those in the ‘different’ image condition were familiar with a greater number of the target faces (*M* = 33.1%, s.d. = 11.3) than those in the ‘same’ image condition (*M* = 20.8%, s.d. = 6.9), *t*_43.51_ = 4.77, *p* < 0.001, *d* = 1.35, BF_10_ = 825.

**Figure 4.**
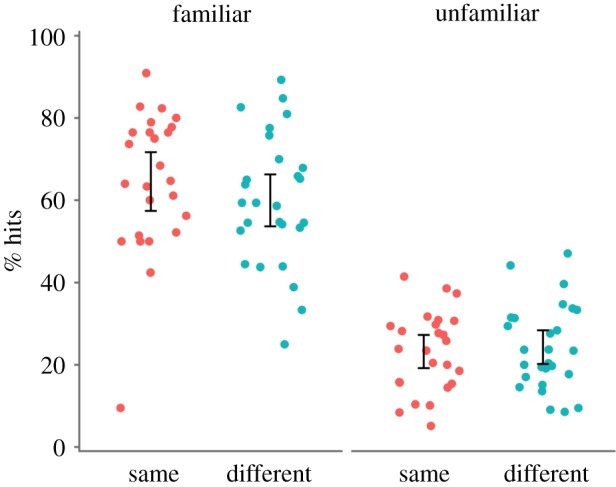
Individual data for correct detection of target repeats by familiarity and group in a subset of participants who were equated for performance on vigilance repeats. Dots are jittered horizontally to improve visibility. In both groups, detection was better for familiar than unfamiliar faces. Error bars show 95% CI around the mean.

The results for the analysis of hit rates are shown in figure 4. We found a main effect of familiarity, *F*_1, 50_ = 353.01, *p* < 0.001, $\eta_{p}^{2}=0.876$, BF_10_ > 1000, but not image, *F*_1, 50_ = 0.30, *p* = 0.585, $\eta_{p}^{2}=0.006$, BF_10_ = 0.315. Crucially, the lack of interaction between familiarity and image remained, *F*_1, 50_ = 1.90, *p* = 0.174, $\eta_{p}^{2}=0.174$, BF_10_ = 0.606. Detection of unfamiliar faces was unaffected by image type, *t*_49.98_ = 0.38, *p* = 0.705, *d* = 0.11, BF_10_ = 0.295, nor was detection of familiar faces, *t*_48.76_ = 0.99, *p* = 0.325, *d* = 0.28, BF_10_ = 0.419. Therefore, even when both groups performed equally on vigilance trials, familiarity with the target face improved detection similarly regardless of whether the repeated face was the same or different.

### 3.2. Exploratory analyses

#### 3.2.1. More specifics of interactions

While the interaction between familiarity and image type in the main analysis was not significant, Bayes factors did not provide strong support for the null hypothesis. But, because the Bayes factor for this effect was calculated without assuming the direction of the interaction, and because the data showed a pattern opposite to our prediction (and what we found in the pilot study), this value understates the true evidence against our registered hypothesis. If we specifically test the hypothesis that unfamiliar faces will be more affected by changes in image by specifying a one-sided test, Bayes factors show strong support for the null hypothesis, BF_10_ = 0.079. This analysis provides additional evidence that our findings are inconsistent with our registered predictions.

#### 3.2.2. d’

**Figure 5.**
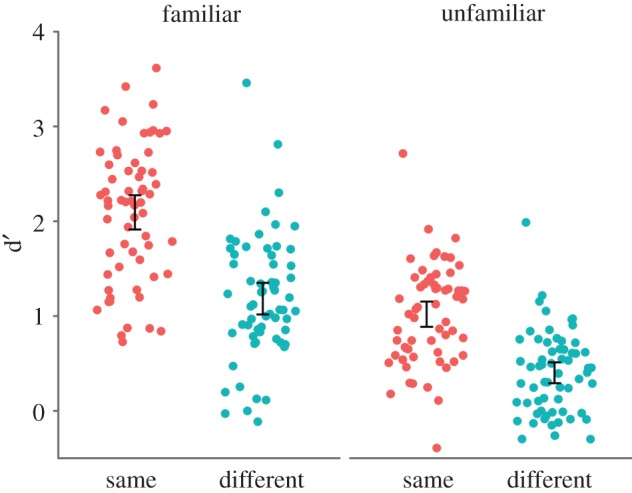
Individual data for d’ by familiarity and group. Dots are jittered horizontally to improve visibility. Participants were more sensitive to repeats of familiar faces than unfamiliar faces in both groups, and more sensitive to repeats in the ‘same’ rather than in the ‘different’ group. Error bars show 95% CI around the mean.

Because we asked participants to indicate their familiarity with 100 of the filler faces, we were able to calculate false alarms (and hence d’) for familiar and unfamiliar faces. These results are presented in table 3 and figure 5. Participants were more sensitive to repeats of familiar (*M* = 1.64) than unfamiliar faces (*M* = 0.71), reflected by the main effect of familiarity, *F*_1, 122_ = 272.79, *p* < 0.001, $\eta_{p}^{2}=0.691$, BF_10_ > 1000. There was also a main effect of image, *F*_1, 122_ = 71.34, *p* < 0.001, $\eta_{p}^{2}=0.369$, BF_10_ > 1000, as repeat detection was better for the ‘same’ (*M* = 1.29) than the ‘different’ (*M* = 0.60) images. There was also an interaction between familiarity and image, *F*_1, 122_ = 6.79, *p* = 0.010, $\eta_{p}^{2}=0.053$, BF_10_ = 3.96. Participants were more sensitive with ‘same’ than ‘different’ images for both unfamiliar faces, *t*_117.79_ = 7.14, *p* < 0.001, *d* = 1.29, BF_10_ > 1000, and familiar faces, *t*_121.22_ = 7.39, *p* < 0.001, *d* = 1.34, BF_10_ > 1000, but this difference was greater for familiar (*M* = 0.93) than unfamiliar faces (*M* = 0.69).

**Table 3.** Group mean (s.d.) d’ and false alarm rates for detection of familiar and unfamiliar targets in the repeat detection task.

	d’		% false alarms	
group	familiar faces	unfamiliar faces	familiar faces	unfamiliar faces
same	2.09 (0.71)	1.02 (0.53)	11.2 (10.1)	9.9 (8.6)
different	1.18 (0.66)	0.40 (0.43)	14.2 (12.1)	9.3 (9.0)

**Figure 6.**
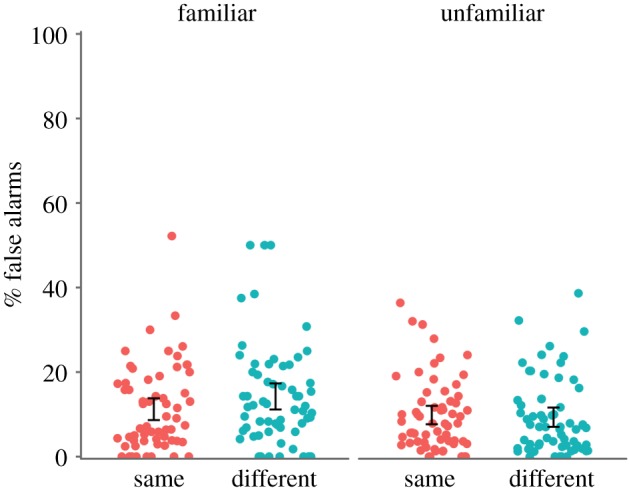
Individual data for false alarms by familiarity and group. Dots are jittered horizontally to improve visibility. Familiar faces were incorrectly detected more when in the ‘different’ group. Error bars show 95% CI around the mean.

The finding of an interaction between familiarity and image with d’ but not hit rates can be explained by differences in false alarm rates (figure 6). False alarms were more common to familiar (*M* = 12.7%) than unfamiliar faces (9.6%), *F*_1, 122_ = 16.60, *p* < 0.001, $\eta_{p}^{2}=0.120$, BF_10_ = 205. There was no effect of image type, *F*_1, 122_ = 0.59, *p* = 0.444, $\eta_{p}^{2}=0.005$, BF_10_ = 0.250. However, there was an interaction between familiarity and image, *F*_1, 122_ = 5.25, *p* = 0.024, $\eta_{p}^{2}=0.041$, BF_10_ = 2.01, such that false alarms were greater for familiar than unfamiliar faces when the images were ‘different’, *t*_61_ = 4.28, *p* < 0.001, BF_10_ = 312, but there was no difference for ‘same’ images, *t*_61_ = 1.33, *p* = 0.188, BF_10_ = 0.322. Although this effect on false alarms was small, when combined with the effects on hit rate, it leads to the interaction found in d’.

#### 3.2.3. Familiarity rating

Participants also rated their familiarity with each target face on a 7-point scale. This rating allows us to assess the effects of familiarity on repeat detection of faces with a more continuous scale, in addition to the dichotomous familiar/unfamiliar scoring by independent coders. To do this, we fit a binomial mixed-effects model predicting hit rates for target repeats. Fixed effects were included for self-reported familiarity, as well as the main effects for and interaction between coder-scored familiarity and image condition (similar to the models used for hit rate and d’). We also fit random intercepts for each participant by coder-scored familiarity (subject effects) and target image identity (item effects). The significance of fixed effects was determined through comparison of nested models (see the electronic supplementary material, table S3 for detailed model comparisons).

**Figure 7.**
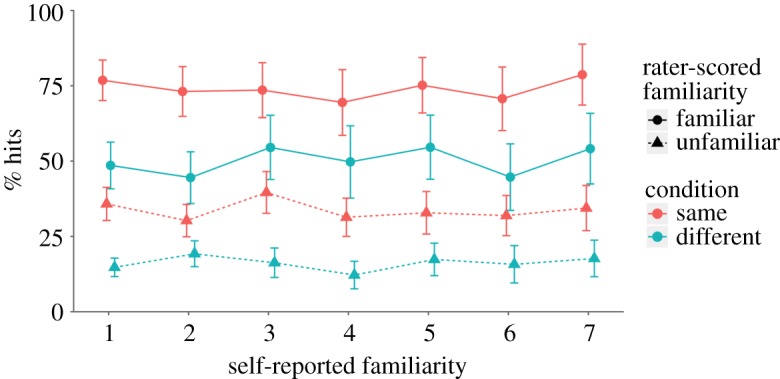
Group average data for correct detection of target repeats by group, coder-scored familiarity and self-reported familiarity. Dot position is adjusted to improve visibility. Self-reported familiarity was unrelated to detection performance. Error bars show 95% CI around the mean.

Notably, there was no effect of self-reported familiarity, $\chi_{1}^{2}=0.19$, *p* = 0.660, suggesting that hit rates in each condition were independent of participants' self-reported familiarity with the target faces. Additionally, this model was able to replicate the previous analysis on hit rate: there was a main effect of coder-scored familiarity, $\chi_{1}^{2}=179.73$, *p* < 0.001, and of image, $\chi_{1}^{2}=42.39$, *p* < 0.001, but no interaction between coder-scored familiarity and image, $\chi_{1}^{2}=0.17$, *p* = 0.676. These data are shown in figure 7, which clearly demonstrates that self-reported familiarity with the target faces was not predictive of actual repeat detection performance. Self-reported familiarity did not differ when coders scored the response as familiar (*M* = 3.11, s.d. = 0.91) and unfamiliar (*M* = 3.16, s.d. = 0.93), *t*_123_ = 1.33, *p* = 0.185, *d* = 0.24, BF_10_ = 0.237. In fact, participants gave numerically higher ratings of familiarity when their responses were scored as unfamiliar, providing further evidence against the ability of self-reported familiarity to influence detection performance.

#### 3.2.4. Relationship between face knowledge and repeat detection

There were substantial participant differences in the knowledge of target and filler faces (*M* = 26.0%, range = 8.5–73.0%). To assess whether greater face knowledge improved detection performance, we correlated participants’ familiarity score with their d’ in the repeat detection task. A participant's ‘face knowledge’ was defined as the percentage of faces they recognized in the familiarity check. We ran separate analyses for familiar and unfamiliar faces because familiar faces had higher overall d’ (figure 5). Greater face knowledge was related to better detection of familiar faces, *r*_122_ = 0.204, *p* = 0.023, although Bayes factors provided inconclusive evidence for this relationship, BF_10_ = 2.05. A similar relationship was found between face knowledge and detection of unfamiliar faces, *r*_122_ = 0.258, *p* = 0.004, BF_10_ = 8.99.

These relationships appear to be driven by a reduction in false alarms as face knowledge increases, rather than greater hit rates, whereas the relationship between face knowledge and hits was not significant for either familiar, *r*_122_ = 0.025, *p* = 0.786, BF_10_ = 0.198, or unfamiliar faces, *r*_122_ = 0.106, *p* = 0.243, BF_10_ = 0.357; a negative relationship was found between face knowledge and false alarm rates for both familiar, *r*_122_ = −0.252, *p* = 0.005, BF_10_ = 7.44, and unfamiliar faces, *r*_122_ = −0.188, *p* = 0.037, although Bayes factors were inconclusive for the latter correlation, BF_10_ = 1.42. Collectively, these data suggest that greater face knowledge (indexed by greater rates of identification in the familiarity check) improved discrimination of familiar and unfamiliar targets from fillers in the repeat detection task.

## 4. Discussion

Theories of face recognition propose that familiar and unfamiliar faces are represented differently in memory: unfamiliar faces are said to rely on *pictorial codes* (the visual details of a specific face image), while familiar faces are thought to rely on *structural codes* (a rich and robust representation of a face generated by averaging across multiple exposures to the same face) [11,24,25]. The robust representations of familiar faces ought to facilitate recognition because they filter out the idiosyncratic information unique to single exposures to a specific face. In this registered report, we examined this proposal with a repeat detection task—an intensive and demanding experimental paradigm more akin to face recognition in real life [36–38]. We had three predictions. First, we predicted better detection of familiar faces. Second, we predicted better detection across ‘same’ images. Third, we predicted an interaction between familiarity and image type. Specifically, we expected stable detection of familiar faces regardless of repeated image, but poorer detection of unfamiliar faces when the repeat involves different images.

As expected, detection was superior when participants were familiar with the target face, and when the face image they had to detect was identical to the initial presentation. Consistent with previous research [26,33], these findings indicate that we are better at recognizing faces which we have had greater exposure to, and that recognizing faces across same images is easier than recognizing them across different images. These results advance the literature by showing that these findings hold in a study with registered data collection and analytic protocols. However, our prediction of interaction between familiarity and image type was not supported. Instead, we found that repeat detection of faces, regardless of familiarity, was poorer across different images. This result is at odds with prior work showing the robustness of familiar face recognition to a wide range of image changes (e.g. lighting and viewpoint) [26–29].

What explains the discrepancy between our finding and those of past studies showing that familiar face recognition is robust to image changes? One possibility is task differences. Unlike many studies that lasted for up to 20 min and used between 30 and 150 face stimuli [2,12,26,33], our task had participants remaining vigilant and paying attention to more than 1000 face images for almost 1 h. If correct, our finding suggests that the robustness of familiar face recognition may falter under more demanding tasks. Future research could vary the parameters of the task (stimulus load, presentation duration, time between repeats, etc.) to further characterize the specific conditions under which familiar face recognition is no longer robust. Another possibility is that because most participants were unfamiliar with a majority of the faces (the average participant only knew about a quarter of the celebrities), they might have relied primarily on a pictorial strategy throughout the experiment. The advantage for familiar over unfamiliar faces might then be due to participants’ simply responding more often to faces they are familiar with, which is consistent with greater false alarms to familiar faces (3.1% increase), although familiar faces also produced greater hits (36.4% increase). This account assumes that familiar face recognition can rely on both structural and pictorial codes, depending on task requirement.

In addition to the registered results, we conducted two exploratory analyses. In one analysis, we found that participants' self-rated familiarity with target faces (as opposed to our scoring/coding of their verbal responses) did not predict how well they detected those faces. This finding accords with recent studies showing that people's judgements about various aspects of face recognition are not related to actual performance [38,48], although it is possible that our participants' notions of familiarity are idiosyncratic because our instruction did not specify what counts as ‘familiar’ (i.e. one participant might have rated a face as ‘highly familiar’ having only seen it a few times, whereas another may need to have seen the face many more times before making the same judgement). A second exploratory analysis revealed that participants who knew more celebrities were more sensitive at the task (as measured by d’). This relationship holds regardless of familiarity and image conditions; participants with greater face knowledge were less likely to mischaracterize a new face as a repeat, even when that face was unfamiliar to them. One explanation might be that both familiar and unfamiliar faces rely on common perceptual representations, such as those proposed by face space [49,50] or holistic [1,51] accounts, the only difference being that familiar faces trigger connections with visual and semantic long-term memory.

Our ability to identify an interaction between familiarity and image type might have been limited by potential floor effects in the unfamiliar/different condition, where performance was particularly low. Our analyses on the subset of participants equated for vigilance and on d’ account for this somewhat, as the floor effects were less apparent (figures 4 and 5). This analysis produced a similar pattern of results to our main analysis. Floor effects aside, it is important to note that we found a large effect of image type for familiar faces. This finding is inconsistent with the notion that familiar face recognition is robust regardless of image. Instead, this finding is more consistent with those from repetition priming studies, where image similarity between the first and repeated presentation of a face predicts participants' ability to recognize the face [40,52]. Our study supports these findings, suggesting that image-level differences contribute to familiar face recognition.

Another potential limitation of our study is fatigue. Participants might have become tired over time because our repeat detection task is long and demanding. This might have led to poorer detection towards the end of the study. However, these effects are unlikely to undermine our findings. Although target trials are more likely later in the repeat detection task (because of the various time lags between repeats), this should be equally true in all conditions, yet we still found a large difference in performance across conditions (from 16% in the unfamiliar/different condition to nearly 75% in the familiar/same condition). Similarly, participants might have responded less reliably in the post-experimental familiarity check. This would lead us to underestimate participants’ true level of familiarity (which could mask potential differences between familiar and unfamiliar faces), yet we still obtained a main effect of familiarity.

Finally, familiar face recognition might rely not only on visual information, but also on semantic knowledge associated with the face (e.g. name and occupation). Because semantic information is only available to familiar faces, they are better detected overall than unfamiliar faces. However, detection still makes use of visual codes, which explains why we see better performance with same rather than different images. This interpretation suggests an important role of semantic knowledge in studies of familiar versus unfamiliar face recognition, an important area for future work.

In summary, this study is the first to investigate the robustness of familiar face recognition with an intensive repeat detection task. The combination of *a priori* power analysis, large sample size, extensive stimulus set and registered protocols makes the results particularly robust. The study demonstrates that familiar faces are memorized better than unfamiliar faces, and that recognition is better when faces are repeated using the same image. However, it suggests that while familiar face recognition benefits from structural codes not available to unfamiliar faces, the benefit may have limits. Finally, it illustrates the value of the repeat detection task as an experimental paradigm that can shed new light not only on familiar and unfamiliar face recognition, but also on other theoretical issues in the face-processing literature.

## Supplementary Material

Pilot study

## Permission to carry out fieldwork

No permissions were required prior to conducting this research.

## Data accessibility

Our data and analysis scripts are available on OSF at https://osf.io/6p2k4/. Additionally, a frozen version of the repository (including data and code from the pilot study), registered prior to conducting the registered study, is available at https://osf.io/j35xf/.
