# Supplementary material for: How robust is familiar face recognition? A repeat detection study of more than 1000 faces
Source: R Soc Open Sci. 2018 May 30;5(5):170634. doi: 10.1098/rsos.170634 (PMC5990823; doi:10.1098/rsos.170634)
Supplement: Pilot study [file rsos170634supp1.docx]

**How robust is familiar face recognition? A repeat detection study of >1,000 faces**

**Supplementary Materials**

Angus F Chapman*^1,2^, Hannah Hawkins Elder*^1^, & Tirta Susilo^1,3^

^1^School of Psychology, Victoria University of Wellington

^2^Department of Psychology, University of California San Diego

^3^ARC Centre of Excellence in Cognition and its Disorders

*Co-first authors

*Royal Society Open Science* (registered report)

Correspondence: Tirta Susilo, School of Psychology, Victoria University of Wellington, PO Box 600, Wellington 6040, New Zealand. Email: [tirta.susilo@vuw.ac.nz](mailto:tirta.susilo@vuw.ac.nz)

**Pilot Study**

**Method**

Except where noted, our method in the pilot study was the same as in the registered study.

**Participants.**

Sixty-four first-year psychology students at Victoria University of Wellington participated for course credit. They were randomly assigned to the ‘same’ image (n = 31) or ‘different’ image (n = 33) conditions. One participant in the different image condition was excluded from analyses because s/he did not respond during the repeat detection task. Eight participants (three in ‘same’ condition; five in ‘different’ condition) did not complete the familiarity task because of a technical error, so their data was excluded. Our sample therefore consisted of 55 participants.

**Procedure.**

**Familiarity task.** In the pilot study, participants identified only the 100 target faces, and did not provide subjective familiarity ratings. All non-blank responses (n = 1533) were scored by two coders (κ = .951) and a third coder resolved any disagreements.

**Technical issue.** A programming error in the familiarity task prevented us from assessing the effects of familiarity as completely as we had hoped. All participants were shown 100 faces, but they were drawn out of the entire target set of 200 images (two images for each face). This means some participants saw some faces twice and didn’t see others at all. On average, participants saw 75.32 unique faces (*SD* = 2.57, range = 70-82). Familiarity for faces that participants saw twice was scored as the higher of the two responses. This error was fixed for our registered study.

Pilot data and analysis scripts are available at [osf.io/6p2k4/](file:////Users/Angus/Dropbox%20(Personal)/VUW/RA%20Work/Tirta/Chapman17RepeatDetection/Stage%202/osf.io/6p2k4/%3fview_only=c15990c96bc0422e984dc804451355b6)

**Results**

**Exclusions.**

Nine participants (3 in the ‘same’, 6 in the ‘different’ groups) were excluded for low familiarity, and one participant in the ‘same’ condition was excluded for poor performance on vigilance repeats. The final sample consisted of 45 participants (21 in the ‘different’, and 24 in the ‘same’ condition).

**Data checks.**

To ensure that data from the familiarity and repeat detection tasks were valid for further analysis, we conducted four checks. First, in the familiarity task, participants identified a reasonable number of faces (*M* = 30.4%, *SD* = 12.6, range = 10.7-55.8%), which did not differ between groups, *t*(42.90) = 0.84, *p* = .405, *d*_s_ = 0.26, BF_10_ = 0.39. Second, in the repeat detection task, hit rates for vigilance repeats (*M* = 46.4%, *SD* = 19.6) was higher than false alarms for fillers (*M* = 12.5%, *SD* = 9.8), *t*(44) = 10.98, *p* < .001, *d_z_* = 1.64, BF_10_ > 1000. This shows that performance on vigilance trials was not simply due to participants responding at random. Third, vigilance detection was greater with ‘same’ than ‘different’ images, *t*(41.58) = 5.85, *p* < .001, *d_s_* = 1.78, BF_10_ > 1000, consistent with previous literature (Armann et al., 2016; Bainbridge, 2017; Bruce, 1982). Four, false alarms for fillers did not differ between groups, *t*(42.72) = 0.55, *p* = .582, *d_s_* = 0.17, BF_10_ = 0.33, showing that false responses to *fillers* was unaffected by changes to the *target* images. *Table S1* contains group means and SDs for data checks; *Figure S1* plots individual data.

| Group | Familiarity task |  | Repeat detection task | |
| --- | --- | --- | --- | --- |
|  | % Faces identified |  | Vigilance | Fillers |
| Same | 29.0 (13.6) |  | 58.3 (17.0) | 13.3 (10.8) |
| Different | 32.1 (11.3) |  | 32.8 (12.2) | 11.7 (8.7) |

*Table S1.* Group mean (SD) percentages of faces identified in the familiarity task, hit rates for vigilance repeats, and false alarms for fillers in the repeat detection task.

**
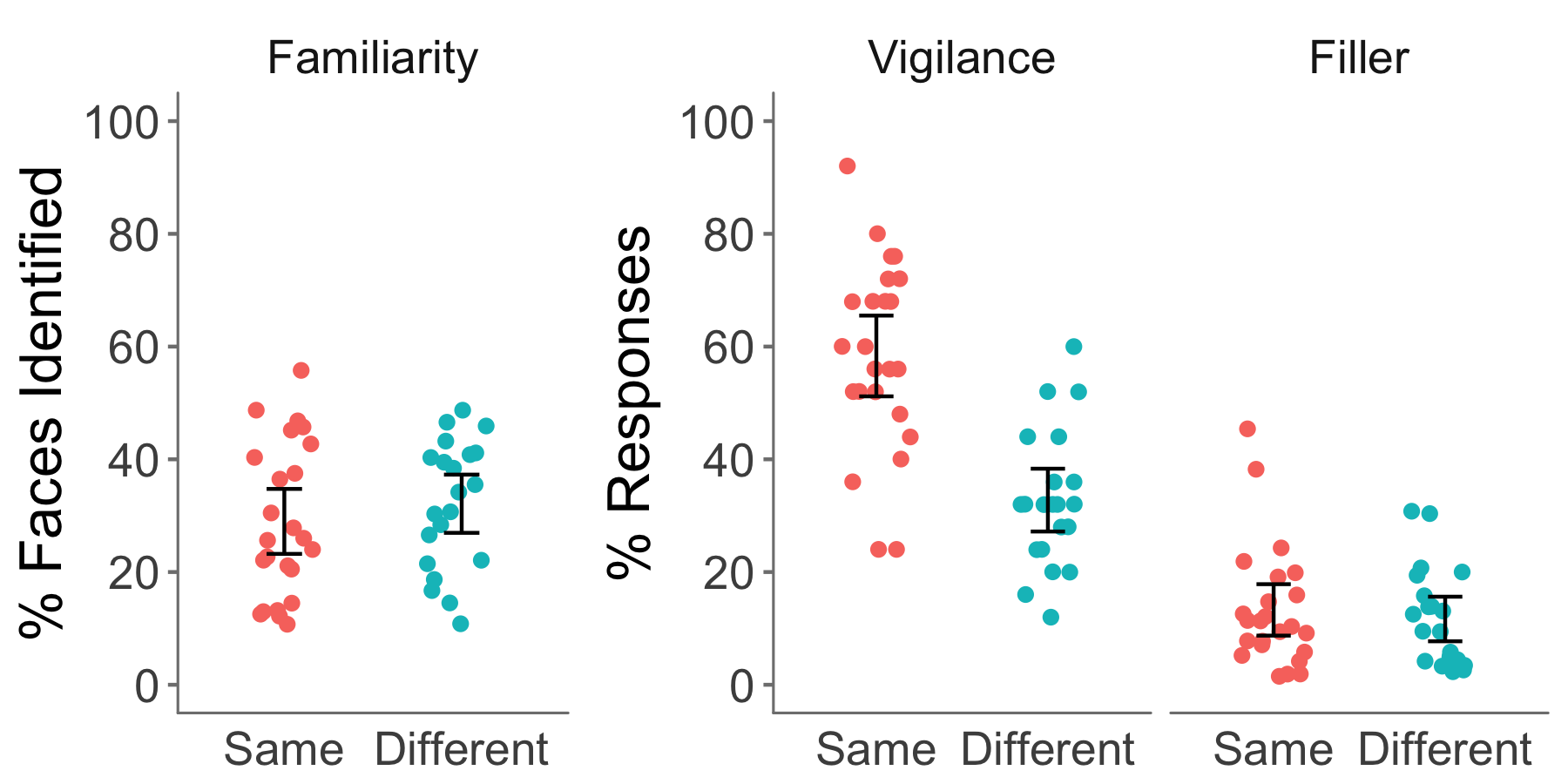
**

*Figure S1*. Individual data for data checks. Dots are jittered horizontally to improve visibility. Left panel shows the number of familiar faces is comparable across groups. Right panel shows that hit rates for vigilance repeats is higher for ‘same’ than ‘different’ groups, whereas false alarms for fillers are similar. Error bars show 95% CI around the mean.

**Main analysis.**

We compared detection hit rates in each group for target faces that participants reported as familiar and unfamiliar. Results are presented in *Table S2* and *Figure S2.* Familiar faces were detected more accurately (*M* = 62.1%) than unfamiliar faces (*M* = 28.0%), reflected by the main effect of familiarity, *F*(1, 43) = 256.57, *p* < .001, $\eta_{p}^{2}$= .856, BF_10_ > 1000. There was also a main effect of image, *F*(1, 43) = 6.38, *p* = .015, $\eta_{p}^{2}$= .129, BF_10_ = 3.54, as repeat detection was better for the ‘same’ (*M* = 44.4%) than the ‘different’ (*M* = 32.3%) images. Critically, these main effects were qualified by a familiarity by image interaction, *F*(1, 43) = 5.79, *p* = .020, $\eta_{p}^{2}$= .119, BF_10_ = 2.84. While unfamiliar faces were better detected with ‘same’ than ‘different’ images, *t*(42.77) = 3.76, *p* < .001, *d_s_* = 1.15, BF_10_ = 52.10, familiar faces were detected equally well regardless of image, *t*(41.28) = 0.99, *p* = .327, *d_s_* = 0.30, although Bayes factors provided only weak evidence for this null effect, BF_10_ = 0.44. This result shows that recognition of familiar faces is robust to image change, but recognition of unfamiliar faces is poorer when the target image is different than the initial face.

| Group | % Hits | |
| --- | --- | --- |
|  | Familiar faces | Unfamiliar faces |
| Same | 64.5 (15.9) | 35.5 (14.0) |
| Different | 59.6 (17.0) | 20.4 (13.1) |

*Table S2.* Group mean (SD) hit rates for detection of familiar and unfamiliar targets in the repeat detection task.

**
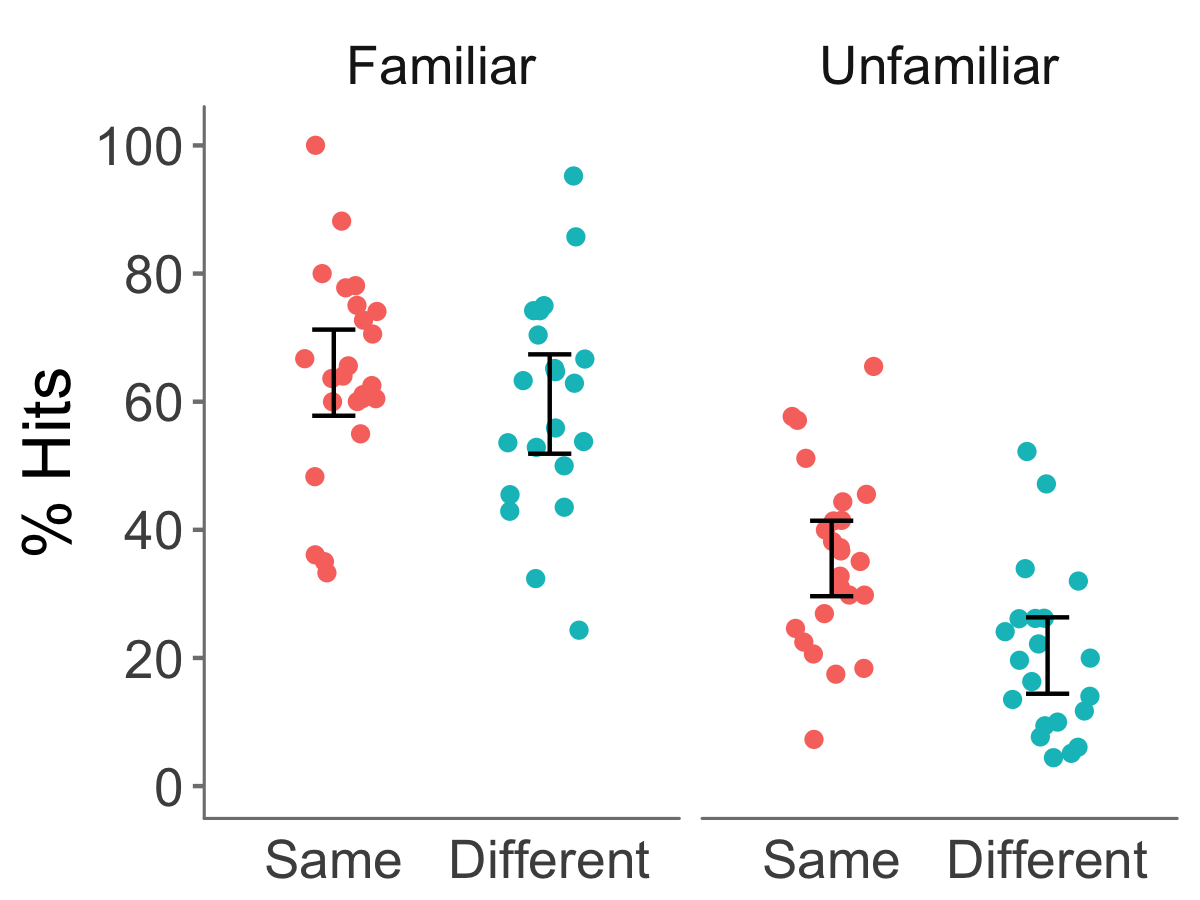
**

*Figure S2*. Individual data for correct detection of target repeats by familiarity and group. Dots are jittered horizontally to improve visibility. Familiar faces were detected equally well by both groups but unfamiliar faces were detected better by ‘same’ than by ‘different’ group. Error bars show 95% CI around the mean.

Can our results be accounted for by the fact that the ‘different’ image condition is more difficult than the ‘same’ image condition? To address this issue, we restricted our analysis to a subset of participants in the ‘same’ condition who performed below the group median on vigilance repeats (≤58.0%, n = 12), and participants in the ‘different’ condition who performed above the group median (≥32.0%, n = 13). This subset was equated on vigilance performance (*M* = 42.2%, *SD* = 10.8), *t*(21.48) = 1.23, *p* = .233, *d_s_* = 0.56, BF_10_ = 0.64, and were familiar with a similar number of faces (*M* = 28.9%, *SD* = 12.0), *t*(20.99) = 1.72, *p* = .101, *d_s_* = 0.79, BF_10_ = 1.07. Results are shown in *Figure S3*. We found a main effect of familiarity, *F*(1, 23) = 179.37, *p* < .001, $\eta_{p}^{2}$= .886, BF_10_ > 1000, but not image, *F*(1, 23) = 0.20, *p* = .656, $\eta_{p}^{2}$= .009, BF_10_ = 0.40. Crucially, the familiarity by image interaction holds, *F*(1, 23) = 9.88, *p* = .005, $\eta_{p}^{2}$= .300, BF_10_ = 9.29. As in the full sample, unfamiliar faces were detected better with ‘same’ (*M* = 32.4%) than ‘different’ images (*M* = 22.7%), *t*(22.84) = 2.07, *p* = .049, *d_s_* = 0.95, BF_10_ = 1.66. In contrast, familiar faces were detected equally well with ‘same’ (*M* = 57.4%) and ‘different’ images (*M* = 63.0%), *t*(22.30) = 0.97, *p* = .344, *d_s_* = 0.44, BF_10_ = 0.51. Although Bayes factors provide only weak evidence in support of these conclusions, the pattern is similar to that in the full sample; even when both groups performed equally on vigilance trials, familiar face recognition remained robust to changes in the image, while unfamiliar face recognition was poorer when the repeated images were different.

**
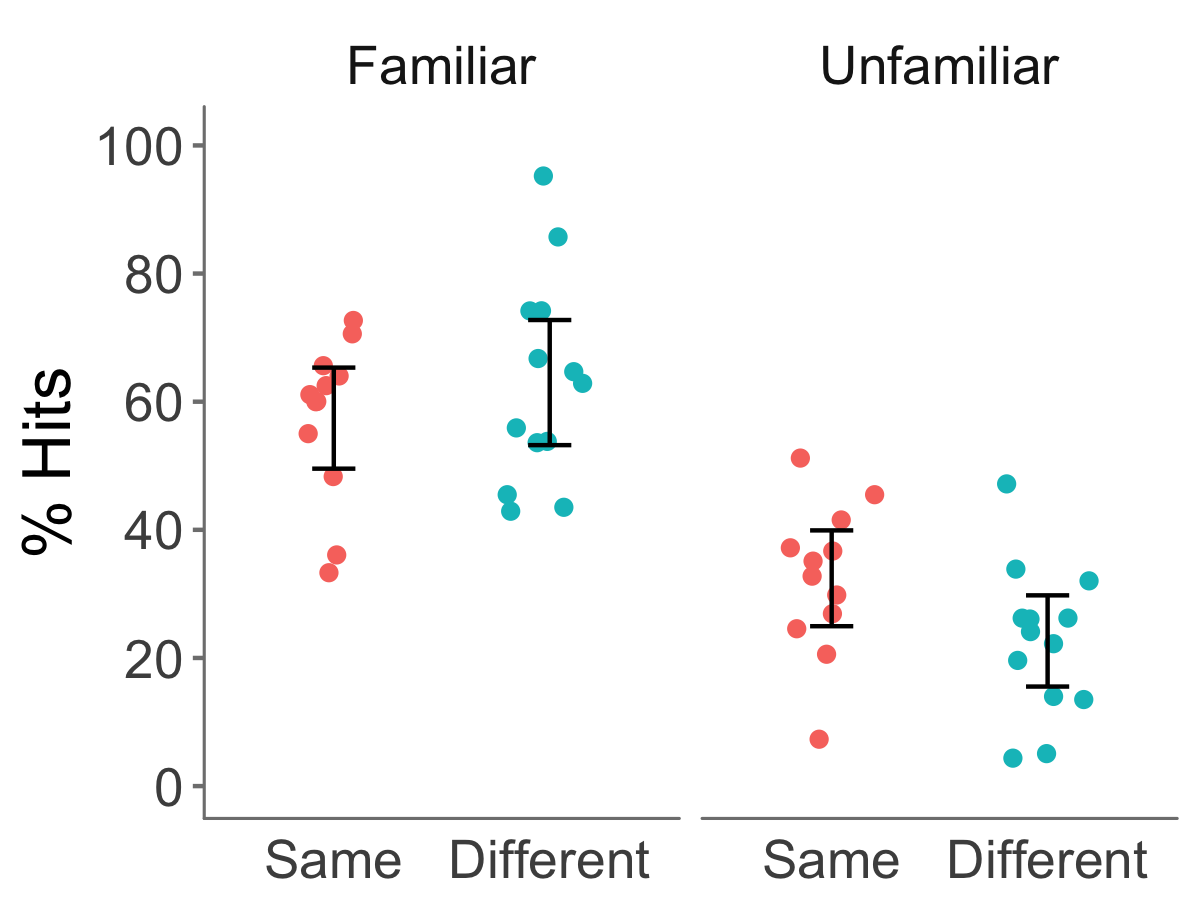
**

*Figure S3*. Individual data for correct detection of target repeats by familiarity and group in a subset of participants who were equated for performance on vigilance repeats. Dots are jittered horizontally to improve visibility. Participants in the ‘different’ group suffered in detection for unfamiliar faces more so than those in the ‘same’ group. Error bars show 95% CI around the mean.

| Model | BIC | df | Δdf | *χ*^2^ | *p* |
| --- | --- | --- | --- | --- | --- |
| Null model | 12957 | 4 |  |  |  |
| + self-reported familiarity | 12966 | 5 | 1 | 0.19 | .660 |
| + coder-scored familiarity | 12796 | 6 | 1 | 179.73 | < .001 |
| + image type | 12763 | 7 | 1 | 42.39 | < .001 |
| + coder-scored familiarity   * image type | 12772 | 8 | 1 | 0.17 | .676 |

*Table S3*. Model selection for the mixed-effects model predicting repeat detection trial-by-trial. Random intercepts for participant, participant by coder-scored familiarity, and target image identity are included in each model. The null model includes only random effects, and no fixed effects.
